# Fault Diagnosis and Classification of Rolling Bearings Using ICEEMDAN–CNN–BiLSTM and Acoustic Emission

**DOI:** 10.3390/s26020507

**Published:** 2026-01-12

**Authors:** Jinliang Li, Haoran Sheng, Bin Liu, Xuewei Liu

**Affiliations:** 1 College of Mechanical and Electronic Engineering, Shandong University of Science and Technology, Qingdao 266590, China; ljl@sdust.edu.cn (J.L.); 202383050052@sdust.edu.cn (H.S.); 2 State Key Laboratory of Geomechanics and Geotechnical Engineering Safety, Institute of Rock and Soil Mechanics, Chinese Academy of Sciences, Wuhan 430071, China; liubin@whrsm.ac.cn

**Keywords:** acoustic emission, ICEEMDAN decomposition, CNN-BiLSTM, rolling bearing fault diagnosis, feature extraction

## Abstract

Reliable operation of rolling bearings is essential for mechanical systems. Acoustic emission (AE) offers a promising approach for bearing fault detection because of its high-frequency response and strong noise-suppression capability. This study proposes an intelligent diagnostic method that combines an improved complete ensemble empirical mode decomposition with adaptive noise (ICEEMDAN) and a convolutional neural network–bidirectional long short-term memory (CNN–BiLSTM) architecture. The method first applies wavelet denoising to AE signals, then uses ICEEMDAN decomposition followed by kurtosis-based screening to extract key fault components and construct feature vectors. Subsequently, a CNN automatically learns deep time–frequency features, and a BiLSTM captures temporal dependencies among these features, enabling end-to-end fault identification. Experiments were conducted on a bearing acoustic emission dataset comprising 15 operating conditions, five fault types, and three rotational speeds; comparative model tests were also performed. Results indicate that ICEEMDAN effectively suppresses mode mixing (average mixing rate 6.08%), and the proposed model attained an average test-set recognition accuracy of 98.00%, significantly outperforming comparative models. Moreover, the model maintained 96.67% accuracy on an independent validation set, demonstrating strong generalization and practical application potential.

## 1. Introduction

With the rapid development of computer and sensor technologies, fault monitoring and diagnosis of rotating machinery have led to the emergence of massive and high-dimensional data [1]. Rolling bearings are critical components in rotating machinery, serving to support and connect rotating elements in modern industry [2]. Bearing performance and lifetime are vital to machine stability; in particular, operating load is a major factor affecting the early failure and reduced service life of bearings [3].

Under prolonged high-load and high-speed operation, bearings are susceptible to typical faults, including inner-ring, outer-ring, rolling-element, cage, and compound faults [4]. Numerous studies have investigated diagnosis methods for various rolling-bearing faults. Vibration-based detection remains the most widely used approach for diagnosing rolling-bearing faults [5]. However, during early fault development or at low rotational speeds, fault signatures in vibration signals are weak and signal energy is mainly concentrated in the low-frequency band. Furthermore, ambient background noise is also often low-frequency, leading to the signal being submerged in environmental noise and making signal separation difficult. In contrast, acoustic emission (AE) is a phenomenon where localized stress concentration in a material rapidly releases energy, generating transient elastic waves [6]. Also known as stress wave emission, AE has a broader spectrum, contains more information, and is predominantly concentrated at high frequencies, which can effectively suppress ambient background noise. Given these circumstances, utilizing acoustic emission detection technology for collecting fault information from rolling bearings has emerged as a new technology and method for rotating equipment fault diagnosis, succeeding the widely used vibration detection methods [7]. Compared to ordinary vibration signals, acoustic emission signals possess characteristics such as greater information content, a wider frequency range, effective exclusion of low-frequency signal interference, and a higher signal-to-noise ratio. Therefore, they can detect rolling bearing faults in a timely manner, ensuring the normal operation of mechanical equipment.

Currently, parameter analysis and waveform analysis are relatively mature acoustic emission signal processing methods [8]. However, when using acoustic emission technology, the collected data volume is immense, making processing time-consuming and fault type identification highly dependent on inspection personnel, thus susceptible to human subjective factors. Consequently, adaptive decomposition methods, notably empirical mode decomposition (EMD), have been applied to acoustic emission (AE) signal processing. EMD adaptively decomposes a signal into a sum of intrinsic mode functions (IMFs) according to local characteristic timescales. The features of each frequency component in the original signal can be analyzed through these IMFs, demonstrating strong denoising and feature extraction capabilities for nonlinear and non-stationary signals [9]. Pandya et al. [10] combined Empirical Mode Decomposition (EMD) with Hilbert–Huang Transform (HHT), utilizing HHT to analyze the IMFs of acoustic emission signals decomposed by EMD, rather than directly applying HHT to the raw signal. The study analyzed five operating conditions: normal bearing, outer-ring fault, inner-ring fault, rolling-element fault, and compound fault. Results indicated that the method improved accuracy in ball-bearing fault diagnosis. However, traditional EMD methods suffer from issues such as high mode mixing and high computational complexity. Colominas et al. [11] proposed an improved complete ensemble empirical mode decomposition with adaptive noise (ICEEMDAN), a newer optimization method for EMD that enhances decomposition efficiency while addressing issues such as residual noise and spurious modes. It is currently being increasingly applied to the analysis of vibration, acoustic emission, and other signals. Wang, Lu et al. [12,13] used kurtosis, correlation coefficients, and other feature indicators to screen signal components decomposed by ICEEMDAN. By combining these with an optimized support vector machine for classification, they achieved good fault recognition results in both rolling bearings and planetary gearboxes. However, research applying this method to the field of noisy signals is still rare.

In bearing fault diagnosis, conventional intelligent approaches typically proceed in two stages: feature extraction followed by classification. Common feature-extraction techniques include the wavelet transform [14] (Wan and Zhang) and the Fast Fourier Transform (FFT) [15] (Safin et al.). Melluso et al. [16] employed discrete wavelet transform to enhance transients and separate components in the residual signal, thereby extracting torque fault signatures associated with internal-combustion engine characteristics under non-ideal operating conditions and mitigating fault-masking effects of adaptive estimators. Once fault features are obtained, appropriate diagnostic techniques are employed to recognize and classify different bearing conditions [17]. Traditional fault-diagnosis methods are generally categorized as data-driven or model-based (mechanism-driven) approaches. Model-based methods use mathematical system representations and detect faults by comparing model outputs with measured signals. However, this approach requires the development of a comprehensive mathematical model, which is often difficult to realize for complex industrial systems, thereby limiting its applicability. In contrast, data-driven methods do not require the construction of complex mathematical models; they rely instead on historical data and statistical analysis. Data-driven methods leverage machine learning to extract fault features directly from data, thereby avoiding the complexity and subjectivity of manual feature engineering. Additionally, data-driven approaches can employ expert systems or fault libraries to perform diagnostic reasoning. This approach offers significant advantages when dealing with complex systems that are difficult to describe using traditional mechanistic models [18,19]. With advances in information technology, a range of intelligent classification algorithms have been developed; tools such as pattern recognition, convolutional neural networks, and deep learning have been applied. The problem of intelligent recognition in rolling bearing fault diagnosis has been further investigated on the basis of feature extraction for different fault modes. After feature extraction, researchers have also conducted studies on the pattern classification of fault types based on acoustic emission signals. Kaewkongka [20] computed parameters from acoustic emission signals, such as energy, peak amplitude, and duration, used the fuzzy c-means clustering algorithm to establish cluster centers, and finally applied a minimum-distance classifier to categorize acoustic emission signals corresponding to different fault types. Pham [21] investigated variable-speed rolling bearings using acoustic emission signals combined with a convolutional neural network model; this model enables automatic classification of bearing fault types with high diagnostic accuracy and can also assess the degree of bearing degradation. Comparison with existing acoustic-emission-based bearing diagnosis methods indicates that the proposed approach demonstrates superior robustness under strong noise interference.

Although traditional methods demonstrate high accuracy in fault detection, most rely on shallow learning algorithms, which cannot achieve autonomous feature learning, adaptive fault feature extraction, or strong generalization capabilities of the model. Convolutional Neural Network (CNN), as one of the important models in the field of deep learning, can combine feature extraction with state classification. Convolutional kernels adaptively learn representative features from signals, reducing reliance on manual feature selection and improving diagnostic accuracy [22,23,24,25]. Li et al. [26] proposed an ensemble deep CNN based on Dempster–Shafer evidence fusion. The method inputs RMS images of FFT features derived from raw time-domain signals to achieve diagnosis under varying operating conditions. Ding et al. [27] proposed a multi-scale feature-mining approach using wavelet-packet energy images and deep CNNs to diagnose faults across varying operating conditions. Zhang et al. [28] developed a CNN that directly ingests raw signals and applies adversarial training strategies to improve diagnosis under varying conditions. Chen et al. [29] embedded a learnable, scale-, and shift-adaptive “Wavelet Kolmogorov–Arnold” kernel into convolution to obtain interpretable spatial features, and used an LSTM augmented with multi-head attention to capture salient temporal dependencies; the method outperformed strong baselines under noise, non-stationarity, and class imbalance while improving interpretability. Hao et al. [30] proposed a multi-sensor approach using one-dimensional convolutional LSTM networks for diagnosis under varying conditions.

However, many bearing-fault methods rely on manual IMF selection or energy-based criteria after EMD-like decompositions, which struggle to consistently emphasize impact-dominant fault information. Moreover, training CNNs directly on raw waveforms yields limited sensitivity to sparse impacts under strong noise and varying conditions, and pure CNNs model long-range, cross-cycle dependencies poorly, limiting generalization and interpretability. To address these issues, we introduce a new IMF selection strategy to enhance sensitivity to impact features in AE signals. On the model side, a CNN learns local temporal patterns and discriminative representations; its time-step features are then fed to a BiLSTM to capture long-range, bidirectional dependencies across cycles, followed by a fully connected classifier.

In summary, we propose an AE-based bearing fault diagnosis method that integrates ICEEMDAN decomposition with a CNN–BiLSTM architecture. We first denoise raw AE signals using wavelet thresholding and assess its effectiveness via signal-to-noise ratio and correlation metrics. To address the inherent non-stationarity of AE and its susceptibility to environmental noise, ICEEMDAN decomposes the signal into intrinsic mode functions at multiple scales. We then apply a kurtosis-maximization criterion to select components most sensitive to impact features, which suppresses random noise and alleviates mode mixing typical of conventional EMD. The selected high-kurtosis IMFs form a feature vector set representing bearing fault types. Feature vector sets corresponding to different fault states are then input into a CNN network to extract the time-domain features of bearing faults. Subsequently, the BiLSTM network layer learns the sequential characteristics of the fault data for classifying and identifying rolling bearing faults. Experimental validation has shown that this method performs well on rolling bearing experimental data under 5 fault modes. The synergy between wavelet thresholding and ICEEMDAN for noise suppression, together with the deep feature learning of CNN–BiLSTM, yields substantial gains in classification accuracy and stability.

The remainder of the paper is organized as follows. Section 2 details the methodology, including AE denoising, ICEEMDAN decomposition, and the CNN–BiLSTM classifier. Section 3 presents the experimental setup and results, comparing the proposed ICEEMDAN–CNN–BiLSTM with baseline models. Section 4 summarizes key findings and contributions. Section 5 concludes and discusses limitations and future directions.

## 2. Methodology Overview

We propose an AE-based bearing fault diagnosis pipeline with four stages: signal pre-processing, ICEEMDAN decomposition, feature extraction, and CNN–BiLSTM classification.

1. Apply wavelet thresholding to suppress background noise and improve the signal-to-noise ratio.
2. Use ICEEMDAN to decompose AE signals into intrinsic mode functions IMFs, and select key IMFs strongly associated with impact features using a kurtosis-based criterion.
3. Employ a CNN to extract discriminative time–frequency features, and a BiLSTM to model temporal dependencies, effectively fusing local patterns with long-range dynamics.
4. Use a terminal classifier to recognize fault types. The method combines adaptive handling of non-stationary components with discriminative deep feature learning, making it suitable for complex operating conditions.

### 2.1. Wavelet Threshold Denoising

When employing acoustic emission (AE) technology for bearing fault diagnosis, it is necessary to first denoise signal data contaminated with background noise. Wavelet denoising, with its advantages of multi-resolution analysis and time-frequency localization, has been successfully applied in the denoising of non-stationary signals.

The signal f(t) undergoes a wavelet transform that concentrates the energy of the useful signal s(t) as much as possible in a few low-frequency components, while the energy of the noise is dispersed across the entire frequency range through successive decompositions. Through multi-layer decomposition, the amplitudes of noise coefficients progressively decrease, while the wavelet coefficients of the useful signal remain unaffected. By applying an appropriate thresholding operation, wavelet coefficients representing noise components are set to zero, while coefficients of the useful signal are retained, thus achieving denoising [31]. Donoho proposed the VisuShrink threshold denoising algorithm, also known as the threshold shrinkage method. The main steps of wavelet threshold denoising are as follows:

1. Select a wavelet basis and determine the decomposition levels. Perform wavelet decomposition on the noisy fault signal to obtain wavelet coefficients $W_{j,k}$.
2. Apply an appropriate threshold and threshold function to quantize the high-frequency coefficients at each level.
3. Reconstruct the denoised signal $\hat{f}$(t) using the thresholded high-frequency coefficients and low-frequency coefficients from each level.

**Figure 1:**
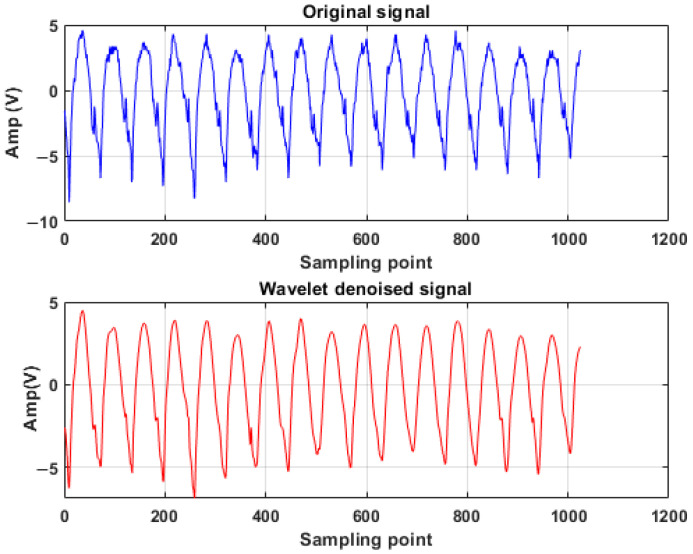
Original signal and wavelet denoised signal.

As shown in Figure 1, the upper panel displays the raw acoustic emission signal of a faulty bearing, while the lower panel presents the resulting AE signal with significantly reduced noise, which is more conducive to subsequent fault feature extraction.

### 2.2. ICEEMDAN Algorithm

CEEMDAN is an improved algorithm of EEMD that effectively suppresses noise issues during reconstruction and enhances decomposition efficiency by adding pairs of positive and negative complementary white noise. However, CEEMDAN decomposition may still exhibit minor residual noise and spurious components. Therefore, this paper investigated the ICEEMDAN decomposition algorithm for extracting acoustic emission signal features, as this method significantly suppresses the residual noise and spurious components present in CEEMDAN decomposition. The ICEEMDAN decomposition procedure is as follows:

1. Add Gaussian white noise to the original signal

(1) $$X_{1}^{\left(i\right)}=x+e_{1}E_{1}\left(w^{\left(i\right)}\right)\left(i=1,2,\ldots ,n\right).$$

where x is the original signal; $e_{1}$ is the expected signal-to-noise ratio for the first decomposition; $w^{\left(i\right)}$ is the i-th added Gaussian white noise; $E_{1}(\cdot )$ represents the first IMF after EMD decomposition.

2. Calculate the first decomposition residual.

(2) $$r_{1}=\left\langle X_{1}^{\left(i\right)}-E_{1}(X_{1}^{\left(i\right)})\right\rangle .$$

where $\left\langle \cdot \right\rangle$ denotes the mean.

3. Calculate the first intrinsic mode component IMF_1_

(3) $${IMF}_{1}=x-r_{1}.$$

4. Estimate the second residual as a series of means and define the second intrinsic mode component IMF_2_

(4) $${IMF}_{2}=r_{1}-\left\langle \left(r_{1}+e_{2}E_{2}(w^{i})\right)\right\rangle .$$

where $e_{2}$ is the expected signal-to-noise ratio for the second decomposition.

5. Calculate the k-th residual r*_k_*

(5) $$r_{k}=\left\langle X_{k}^{\left(i\right)}-E_{k}(X_{k}^{\left(i\right)})\right\rangle .$$

6. Calculate the k-th intrinsic mode component ${IMF}_{k}$

(6) $${IMF}_{k}=r_{k-1}-r_{k}$$

7 Return to step 5 for calculation $r_{k+1}$

**Figure 2:**
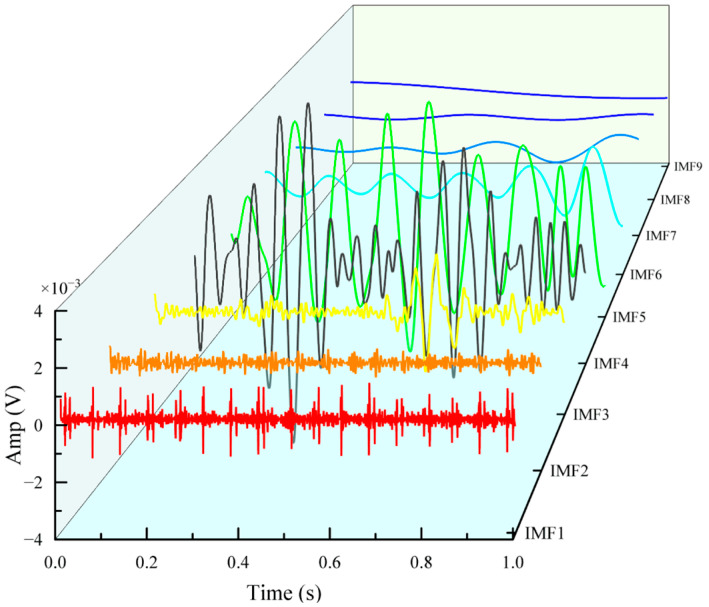
IMF components obtained from ICEEMDAN decomposition.

After wavelet-threshold denoising, the faulty bearing AE signal is decomposed using ICEEMDAN to obtain a series of IMFs; Figure 2 illustrates this process for an inner-ring fault example. From the figure, it can be observed that lower orders correspond to higher frequencies. The kurtosis values of the 9th order IMF components were calculated separately. Based on the kurtosis screening criterion, IMF components containing more original features were identified to prepare for subsequent fault information feature extraction.

### 2.3. CNN-BiLSTM Model for Fault Type Classification

#### 2.3.1. Convolutional Neural Network

**Figure 3:**
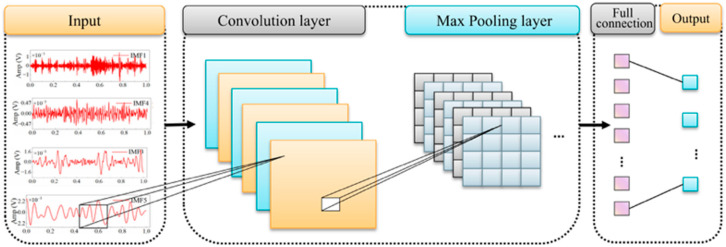
CNN structure diagram.

A convolutional neural network (CNN) is a feedforward model characterized by local connectivity and weight sharing. A typical CNN comprises an input layer, convolutional and pooling layers, fully connected layers, and an output layer [32]. The overall CNN architecture is shown in Figure 3.

Traditional diagnosis pipelines—feature extraction followed by pattern recognition and classification—often fail to exploit temporal dependencies in fault time series, resulting in discontinuities. CNNs can learn discriminative features and improve diagnostic accuracy and efficiency, but standalone CNNs are limited in modeling the temporal dynamics of bearing signals [33]. A bidirectional long short-term memory (BiLSTM) network consists of forward and backward LSTM layers and can exploit both past and future context in one-dimensional time series, thereby improving temporal modeling.

#### 2.3.2. Bidirectional Long Short-Term Memory Network

A Bidirectional Long Short-Term Memory Network (BiLSTM) [34] comprises two LSTM networks operating in opposite temporal directions: a forward LSTM and a backward LSTM.

LSTM is a variant of recurrent neural network (RNN) architecture capable of overcoming issues such as vanishing gradients in RNNs. The primary improvement of LSTM over RNN lies in the addition of three gate structures within the hidden layer *h*, along with an additional hidden state. In BiLSTM, input sequences are fed in both forward and reverse order to two separate LSTM neural networks for feature extraction. The two resulting output vectors are then concatenated to form a new word vector, which serves as the ultimate feature representation for that word. BiLSTM neural networks can handle sequences of variable lengths and can also process batches of sequences with differing lengths. CNN-BiLSTM combines the advantages of CNN in feature extraction with BiLSTM’s ability to capture long-range dependencies when processing sequential data.

**Figure 4:**
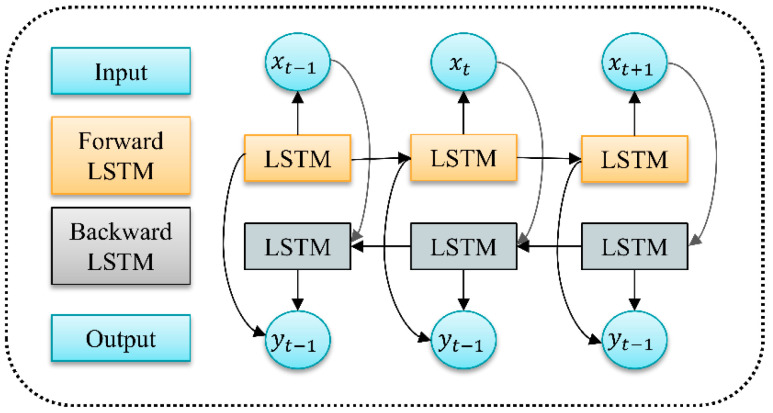
BiLSTM structure diagram.

The BiLSTM structure is shown in Figure 4.

## 3. Experimental Validation

### 3.1. Test Platform and Experimental Procedure Introduction

**Figure 5:**
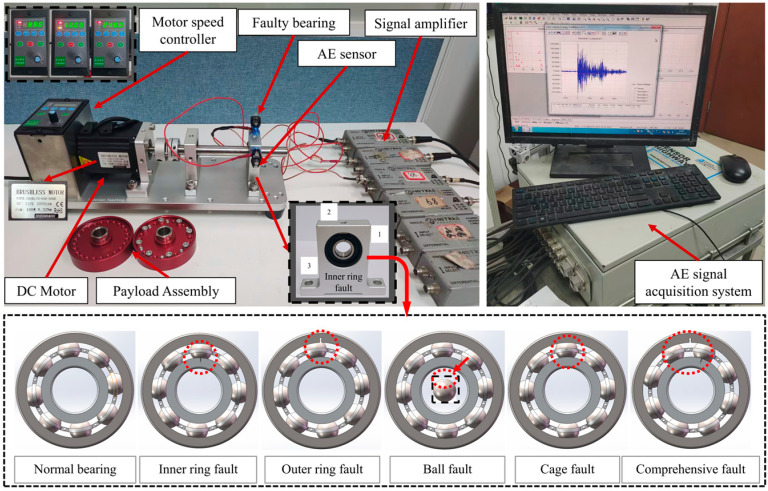
Schematic diagram of integrated mechanical fault simulation test bench and faulty bearing.

Experiments were conducted using the PT300 comprehensive mechanical fault simulation test rig, as shown in Figure 5. This equipment is manufactured by VALENIAN Company in Suzhou, China. This test rig offers advantages such as quick startup, low vibration, low noise, and ease of use, fulfilling all experimental requirements of this study. The signal acquisition equipment included an RS-54A piezoelectric ceramic acoustic emission sensor from Beijing Softland Times Technology Co., Ltd. (Beijing, China), a PAC-compatible AE acquisition card, and preamplifier. The RS-54A AE sensor has a monitoring frequency range of 100 kHz–900 kHz, while the preamplifier provides a gain of 40 dB and a bandwidth of 10.0 kHz~2.0 MHz, serving to enhance the signal-to-noise ratio within the entire system. In experiments, three AE sensors were mounted at designated locations on the bearing housing at positions 1–3 in the figure to ensure optimal signal reception. A dedicated couplant was used to improve sensor-bearing coupling and reduce attenuation. The acquisition bandwidth was set to 1 MHz.

Five 6001 deep groove ball bearings were used in the experiments; their structural parameters are detailed in Table 1. By machining the corresponding positions of normal bearings, they are transformed into target faulty bearings. The five faulty bearings exhibited distinct failure types: inner ring failure, outer ring failure, rolling element failure, cage failure, and combined failure. Acoustic emission signals were collected by replacing different faulty rolling bearing assemblies. During testing, the motor speed of the test platform was controlled at 800 r/min, 1600 r/min, and 2400 r/min, encompassing a total of 15 operating conditions.

### 3.2. Data Processing and Dataset Establishment

#### 3.2.1. Denoising of AE Signals from Faulty Bearings

**Figure 6:**
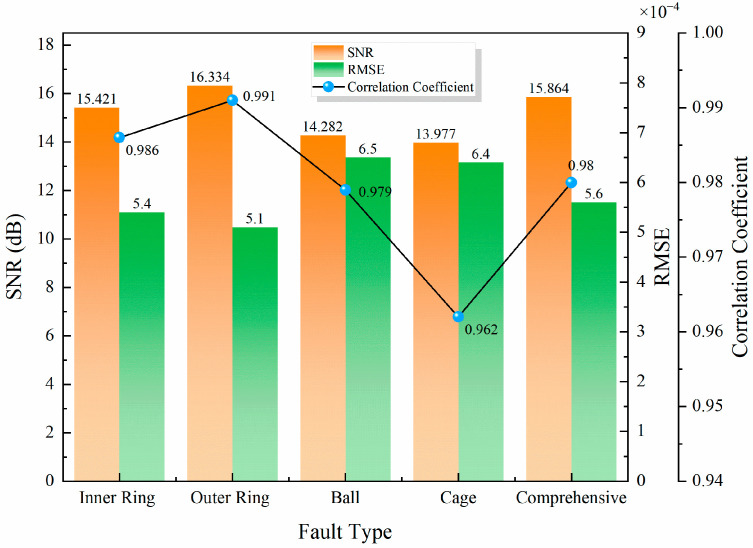
Evaluation coefficients for WTD.

After the experiments, one set of AE signals was taken from each of the five types of faulty bearings. The raw AE signals of the selected faulty bearings were subjected to wavelet threshold denoising. The denoising effectiveness was quantitatively evaluated using three metrics: signal-to-noise ratio (SNR), root mean square error (RMSE), and correlation coefficient. Figure 6 presents the denoising metrics for the five fault types. The results indicate that the average SNR of the denoised signals increased to 15.175 dB, signifying effective noise suppression. The root mean square error (RMSE) was as low as 0.00058, reflecting minimal distortion introduced by the denoising process. Concurrently, the average correlation coefficient between the denoised and original signals reached an impressive 0.979, demonstrating excellent preservation of critical waveform features. These three metrics collectively confirm that the employed wavelet denoising method effectively removes background noise while maximally retaining the transient impulse components indicative of bearing fault conditions, thereby establishing a reliable foundation for subsequent feature extraction and fault identification.

#### 3.2.2. Comparison of EMD, CEEMDAN, and ICEEMDAN Decompositions

**Figure 7:**
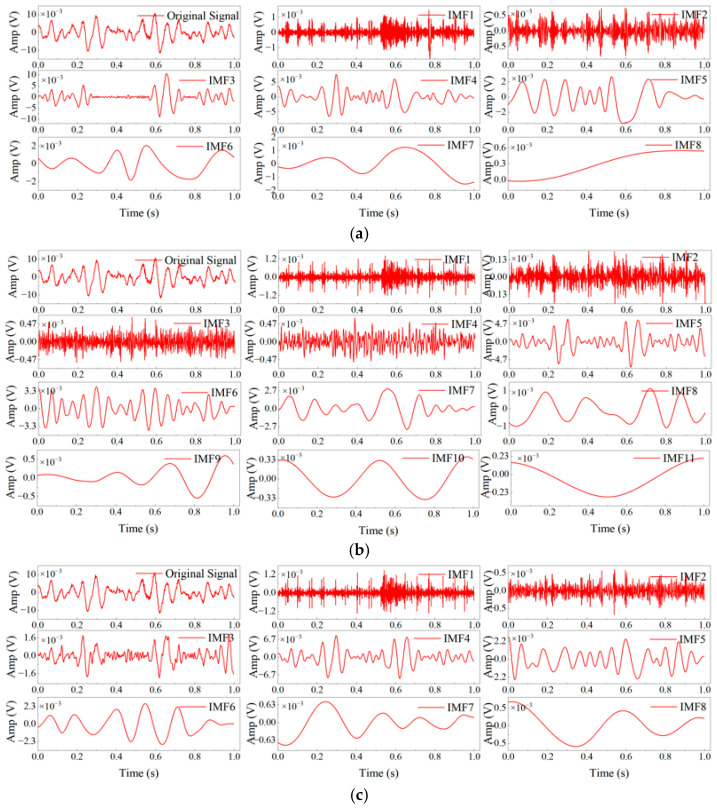
Results of different decomposition methods for ball bearing fault signals: (**a**) EMD decomposition, (**b**) CEEMDAN decomposition, and (**c**) ICEEMDAN decomposition.

This paper employed EMD, CEEMDAN, and ICEEMDAN algorithms, respectively, for feature extraction from denoised acoustic emission signals to verify the performance of these three decomposition methods. One set of wavelet-threshold denoised acoustic emission signals was taken from each of the five faulty bearings and subjected to EMD, CEEMDAN, and ICEEMDAN decomposition, respectively, yielding multiple high-to-low frequency IMF components and a residual component. Due to the excessive amount of data, only the decomposition results of the acoustic emission signals for a rolling element faulty bearing are presented, as shown in Figure 7.

As shown in the figure, comparison of the IMF components obtained by EMD, CEEMDAN, and ICEEMDAN clearly demonstrates the advantages of ICEEMDAN for fault-signal decomposition. For EMD (Figure 7a), its IMF components exhibited pronounced mode mixing: the high-frequency IMFs (IMF1, IMF2) displayed irregular, spiky oscillations while containing multi-scale frequency components, and mid-frequency IMFs (e.g., IMF3) were contaminated by high-frequency signals, preventing effective separation of fault-characteristic frequencies; whereas the IMF components from ICEEMDAN (Figure 7c) showed that the high-frequency component IMF1 oscillated uniformly and singularly, corresponding to the high-frequency fault impact; the mid-to-low-frequency components (IMF2, IMF3) exhibited regular waveforms and well-defined frequency scales, with mode mixing effectively suppressed. Compared with CEEMDAN (Figure 7b), whose IMF components suffer from residual-noise redundancy and spurious modes—for example, excessively large oscillation amplitudes in high-frequency IMFs and abrupt, irregular oscillations in IMF—ICEEMDAN’s IMF components have their residual noise removed, as evidenced by smoother oscillation amplitudes in high-frequency components, continuous waveforms without spurious modes, and better preservation of fault-related information. Moreover, the scale-wise stratification of ICEEMDAN IMFs from high to low frequency is more discriminative; the boundaries between high-frequency impacts, mid-frequency fundamental components, and low-frequency trend features are well-delineated, providing a more accurate modal foundation for subsequent feature extraction.

To better compare the decomposition performance of EMD, CEEMDAN, and ICEEMDAN, the mode-mixing rate of the IMF components produced by each algorithm was calculated. Mode mixing refers to the phenomenon in Empirical Mode Decomposition (EMD) where an intrinsic mode function (IMF) contains signal components from different time scales, or where a single time-scale component is incorrectly distributed across multiple IMFs. This phenomenon degrades the quality and reliability of the decomposition results, thereby affecting subsequent fault diagnosis and feature extraction; hence, a lower mixing rate indicates better decomposition performance.

**Figure 8:**
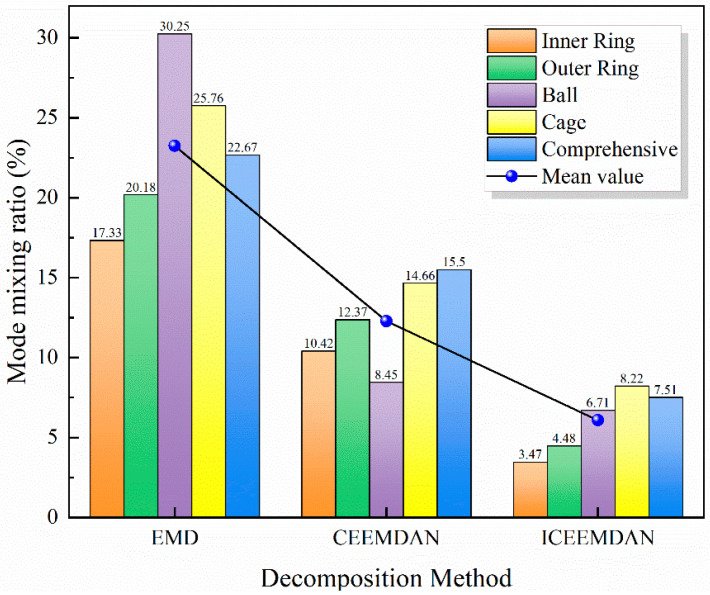
Modal overlap rates for different decomposition methods.

Figure 8 presents the mode-mixing rates for five faulty bearings under the three decomposition methods. Among them, EMD yielded the highest mode-mixing rates, ranging from 17.33% to 30.25%; this is attributable to intrinsic limitations of the EMD algorithm, where mode mixing is common. CEEMDAN, which integrates ensemble EMD with adaptive noise, reduces mode mixing, reported mixing rates ranging from 8.45% to 15.50%. ICEEMDAN achieved the lowest mode-mixing rates—ranging from 3.47% to 8.22%—because it further refines CEEMDAN via more precise noise control and adaptive adjustments, thereby further reducing mode mixing. The figure clearly shows that IMFs obtained by ICEEMDAN have the lowest mode-mixing rates, with an average of only 6.08%, indicating purer IMF components and superior decomposition performance.

ICEEMDAN exhibits significant advantages over CEEMDAN and traditional EMD. By adaptively adjusting the added white noise, ICEEMDAN enhances the algorithm’s robustness to nonlinear and non-stationary signals, enabling more accurate extraction of the signal’s principal components. ICEEMDAN reduces the number of iterations and improves decomposition speed through an improved white-noise generation and selection mechanism. Furthermore, ICEEMDAN performs better at avoiding mode mixing, resulting in purer IMFs with clearer boundaries. The IMFs obtained by ICEEMDAN more closely corresponded to the main components of the original signal, while other IMFs contained fewer mixed components.

#### 3.2.3. Kurtosis Values

Among the series of IMF components generated after ICEEMDAN decomposition of the raw signal, not all components effectively represent the original signal’s characteristics. Therefore, spurious IMF components should be discarded to improve fault-category discrimination. This paper employed the kurtosis index as the screening criterion. To extract salient features from AE signals, we computed the kurtosis of each IMF and selected components with high kurtosis values.

Kurtosis is a statistical measure that characterizes the peakedness of a signal’s amplitude distribution. Its mathematical expression is:

(7) $$k=\frac{{E(x-\mu )}^{4}}{\sigma^{4}}$$

where *x* is the analyzed acoustic emission signal; μ is the mean of signal *x*; σ is the standard deviation of signal *x*; *E* is the mathematical expectation.

Kurtosis is a dimensionless statistic highly sensitive to impact components. For AE-based impact faults, impacts carry key diagnostic information. As a measure of peak sharpness, kurtosis effectively separates impacts from background noise and is well-suited to diagnosing surface damage and early-stage faults. Signals from healthy bearings are approximately Gaussian with kurtosis near 3. Bearing faults often generate impulsive signal components, which increase the kurtosis value. From this, it can be inferred that if the kurtosis value of certain IMFs is greater than 3, it indicates that more fault-related impulsive information is retained in these IMFs after ICEEMDAN decomposition of the original signal. Therefore, a larger IMF kurtosis value implies easier extraction of fault information.

**Figure 9:**
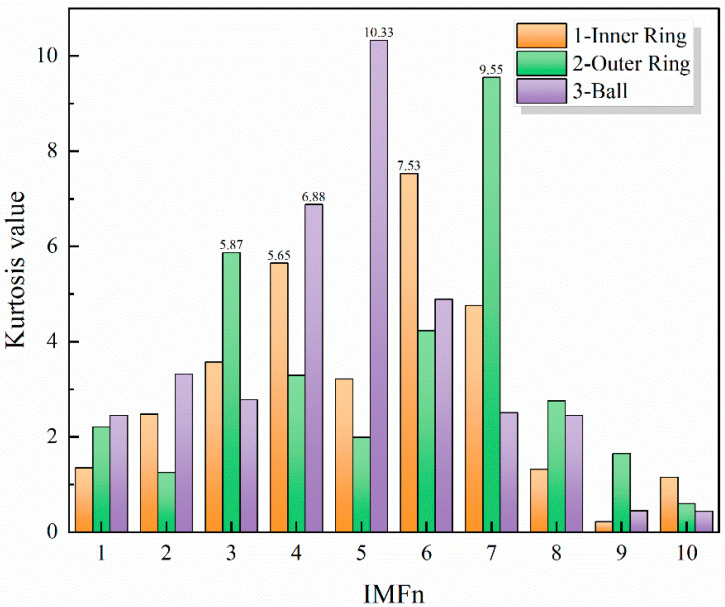
Kurtosis values for different IMF components.

Due to the large volume of data, only partial results are presented. Figure 9 shows the kurtosis values of three signals from inner race faulty bearings, outer race faulty bearings, and rolling element faulty bearings. It is evident that the kurtosis values corresponding to IMF components of different orders vary significantly. Selecting the two components with the largest kurtosis values can lead to more effective extraction of fault information.

Following the above procedure, two components obtained by screening all IMF components using kurtosis values were taken as one sample for a fault type, with a sample length of 2048. The experiment set up 5 fault types and 3 speed levels: 800 r/min, 1600 r/min, and 2400 r/min, which were cross-combined to form 15 independent operating conditions. One set of experimental data was collected for each operating condition, from which 120 effective samples were selected. To ensure generalization, we explicitly separated the training and test data. For each class, 90 samples were used for training and the remaining 30 for validation, with no overlap or shared segments between the two sets.

### 3.3. Model Training and Validation

**Figure 10:**
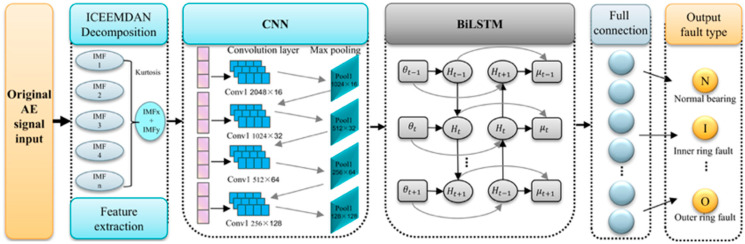
ICEEMDAN–CNN–BiLSTM model architecture diagram.

This paper proposes an ICEEMDAN-optimized decomposition algorithm combined with a CNN–BiLSTM model for rolling bearing fault diagnosis. The flowchart of this method is illustrated in Figure 10.

The proposed network comprises four convolutional layers, four pooling layers, one fully connected layer, one BiLSTM layer, and a final Softmax layer. We trained the model with Adam for 150 epochs using a batch size of 32, an initial learning rate of 0.01, a learning-rate decay factor of 0.01, DropConnect of 0.5, Dropout of 0.5, ReLU activations, and max pooling. Detailed layer settings are reported in Table 2. Additional training hyperparameters include MaxEpochs, InitialLearnRate, LearnRateSchedule, and L2Regularization, which jointly affect optimization and final performance.

The specific steps for bearing fault diagnosis using AE signals with this model are as follows:

1. Signal Preprocessing: The fault bearing AE signals collected from the mechanical fault test rig are organized, segmented, selected, and then subjected to wavelet threshold denoising.
2. Signal Decomposition: The processed AE signals are decomposed into a series of IMF components using the ICEEMDAN algorithm.
3. Signal Reconstruction: The kurtosis value of each IMF component is calculated. IMF components with larger kurtosis values are selected for signal reconstruction, forming feature vectors.
4. Feature Extraction: Time–frequency domain features are extracted using CNN and then fused.
5. Pattern Recognition: The BiLSTM network is utilized to learn sequential features for training and pattern recognition, thereby accomplishing the fault diagnosis of rolling bearing AE signals.

**Figure 11:**
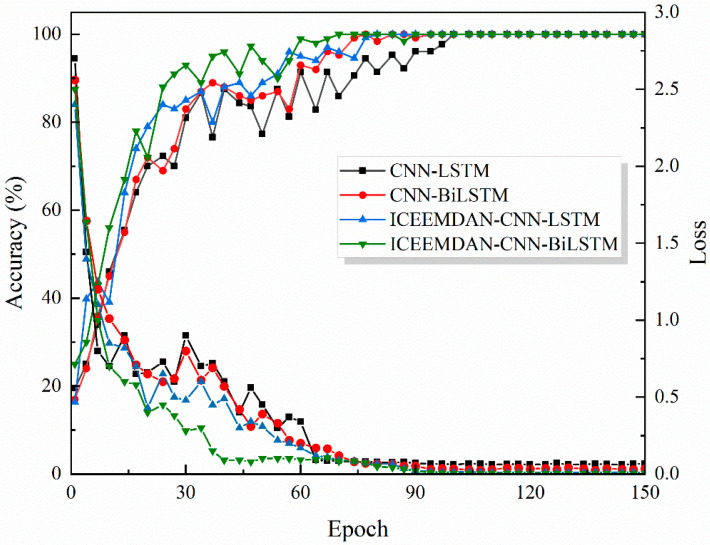
Accuracy and Loss during the Training Process of Four Models.

To validate ICEEMDAN–CNN–BiLSTM performance, we conducted comparative experiments against CNN–LSTM, CNN–BiLSTM, and ICEEMDAN–CNN–LSTM baselines. The same dataset was used for these experiments, with a training set to test set ratio of 3:1. Specific parameters are detailed in Table 3. To ensure fair comparison, all four models used consistent hyperparameters—learning rate, batch size, optimizer, regularization, and maximum epochs—so that the performance differences reflected the architecture. We used Adam with initial learning rate 0.01 and batch size 32. Architectural settings were aligned: all CNNs had four convolutional layers with 32 filters per layer, and BiLSTM layers had 512 units to match model capacity. Limited tuning was applied based on validation: increase Dropout or adjust batch size when overfitting; deepen the network or modify the learning-rate schedule when underfitting. Figure 11 shows the accuracy and batch loss during training for the four models over 150 iterations. From the figure, it can be observed that the proposed ICEEMDAN–CNN–BiLSTM method outperformed the other three models in terms of both accuracy and loss.

**Figure 12:**
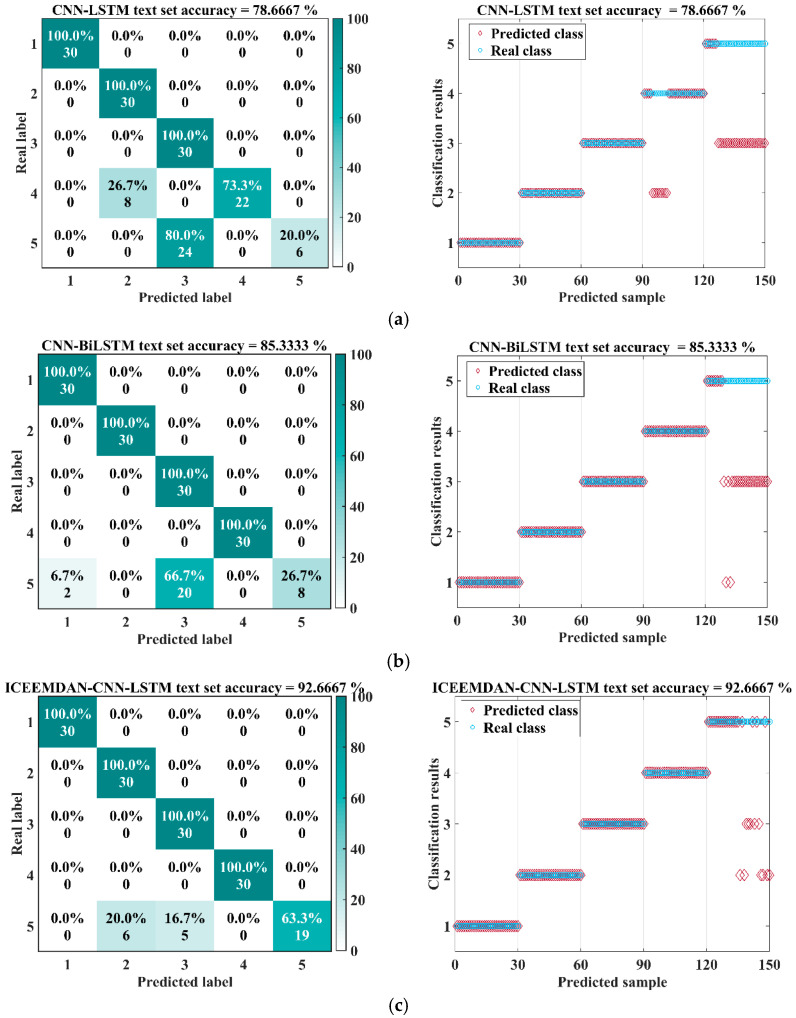

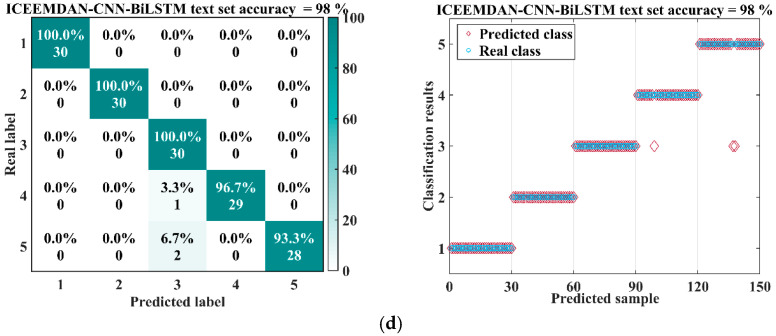
Comparison of Results for Four Models on the Same Dataset: (**a**) CNN-LSTM, (**b**) CNN-BiLSTM, (**c**) ICEEMDAN-CNN-LSTM and (**d**) ICEEMDAN-CNN-BiLSTM.

Table 4 reports the trained models’ results. ICEEMDAN–CNN–BiLSTM performed best, achieving the highest scores on all core metrics: accuracy of 98.00 with the smallest variability in standard deviation ± 0.80 percentage points. Using F1 as a reference, it improved over CNN–LSTM, CNN–BiLSTM, and ICEEMDAN–CNN–LSTM by 19.37, 12.70, and 5.37 percentage points, respectively. Figure 12 presents the confusion matrices and test results for the four models on a common test set. Rows correspond to true labels and columns to predicted labels; types 1–5 denote the inner-ring, outer-ring, ball, cage, and compound faults, respectively. The test set accuracies of the four algorithms showed significant differences. In Figure 12a, the CNN–LSTM model had the lowest test accuracy, only 78.67%. From the confusion matrix, it can be seen that the recognition accuracy for fault type 4 was 73.3%, and for compound fault type 5, the signal recognition rate was only 20%. The recognition accuracy of the CNN–BiLSTM model improved to 85.33%. The bidirectional temporal modeling capability of BiLSTM improved the recognition of type 4 faults, with a 100% recognition rate. However, 66.7% of type 5 fault signals were still misclassified as type 3, indicating that simply optimizing the network structure has limited impact on improving model recognition performance. The recognition accuracy of the ICEEMDAN–CNN–LSTM model further increased compared to the previous two, with the recognition rate improving to 92.67%. ICEEMDAN’s mode decomposition preprocessing improved the recognition rate of type 5 signals to 63.3%, demonstrating the role of fault signal preprocessing in fault feature extraction. The test accuracy of the ICEEMDAN–CNN–BiLSTM model reached an impressive 98%, significantly higher than the other three models, making it the optimal result. Only one sample in type 4 was misclassified as type 3, and two samples in type 5 were misclassified. This reflects the superiority of the proposed method, demonstrating that maximum utilization of fault features and temporal information can be achieved through ICEEMDAN preprocessing and BiLSTM. The results indicate that augmenting CNNs with BiLSTM surpasses uni-directional LSTM, demonstrating the value of bidirectional temporal modeling; ICEEMDAN-based preprocessing provides additional gains; together they yield complementary benefits, improving both accuracy and robustness.

### 3.4. Model Application

**Figure 13:**
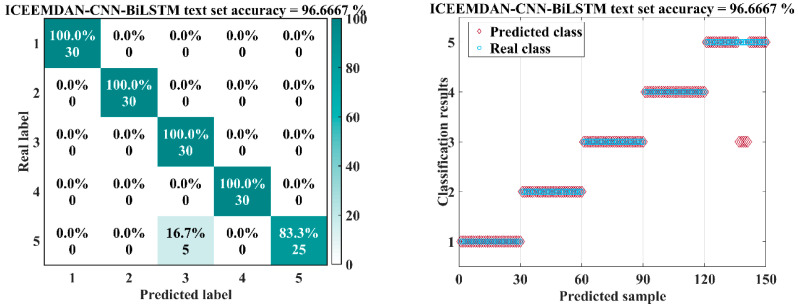
Validation set model results: confusion matrix and validation results.

To further assess the effectiveness of the ICEEMDAN–CNN–BiLSTM model for rolling bearing fault diagnosis, the author selected 30 new samples for each bearing fault type from the data obtained in the experiments conducted in Section 3.1. This selection was used to form a new validation set aimed at evaluating the method’s effectiveness and generalization capability. As shown in Figure 13, the validation results indicate an identification accuracy of 96.67%, with only five samples of type-5 (compound) faults misidentified as type-3 (ball) faults and a runtime of only 10.12 s. All experiments were conducted on a system with an Intel Core i5-12400 CPU, 32 GB RAM, and Windows 11. These findings demonstrate that the model achieves higher accuracy and greater robustness, while the optimized preprocessing of acoustic emission signals further enhances performance and speed.

## 4. Discussion

In this work, a rolling-bearing AE signals fault-diagnosis approach that combines an ICEEMDAN-optimized decomposition algorithm with a CNN–BiLSTM model was proposed. The effectiveness and strong recognition capability of this model was validated through experiments on faulty bearings and comparative model tests. The main conclusions can be drawn as follows:

1. In the ICEEMDAN–CNN–BiLSTM model, raw acoustic emission (AE) signals from faulty bearings are denoised using wavelet thresholding to improve the signal-to-noise ratio. The ICEEMDAN algorithm decomposes the processed signal into multiple intrinsic mode function (IMF) components. Those with higher kurtosis values are selected to represent the original fault signal, thereby reducing the dimensionality of the feature set and lessening the computational load for subsequent fault identification. CNN then extracts and fuses time–frequency domain features from these components. Finally, BiLSTM learns sequential dependencies within the features for model training and pattern recognition, enabling fault diagnosis of rolling bearing AE signals.
2. In the signal preprocessing stage, the wavelet threshold denoising method effectively eliminates background noise while optimally preserving the transient impact components that characterize bearing fault conditions. Following wavelet threshold denoising, the processed signals achieved an average signal-to-noise ratio (SNR) of 15.175 dB, a root-mean-square error (RMSE) as low as 0.00058, and an average correlation coefficient between the denoised and original signals of up to 0.979.
3. The ICEEMDAN decomposition algorithm demonstrated superior performance compared to traditional EMD and CEEMDAN when processing nonlinear and non-stationary acoustic emission signals. By analyzing mode-mixing rates, it was evident that ICEEMDAN achieved the lowest mixing rate, averaging only 6.08%, which was significantly lower than EMD’s 23.24% and CEEMDAN’s 12.28%. This finding validates ICEEMDAN’s distinct advantages in mitigating mode mixing, eliminating residual noise redundancy, and removing spurious modes, thereby yielding purer IMFs with enhanced discriminability across frequency scales. Notably, the high-frequency IMF effectively characterizes fault impacts, while the mid-to-low-frequency components exhibit regular waveforms, providing a more precise modal basis for subsequent feature extraction.
4. The ICEEMDAN–CNN–BiLSTM model exhibits high fault recognition rates and robust performance in practical applications. When compared to the CNN–LSTM, CNN–BiLSTM, and ICEEMDAN–CNN–LSTM models, the proposed model clearly achieved superior recognition performance. In the experimental evaluations, the average recognition accuracy across the test and validation sets reached an impressive 97.33%, demonstrating strong generalization capabilities.

## 5. Conclusions

### 5.1. Summary of Findings

We presented an AE-based bearing fault diagnosis method that combines ICEEMDAN with a CNN–BiLSTM architecture. Experiments across multiple operating conditions showed strong performance, with 98.00% mean test accuracy and 96.67% accuracy on an independent validation set, indicating good generalization and practical potential. ICEEMDAN effectively suppresses mode mixing, while CNN–BiLSTM enables end-to-end fault recognition, significantly outperforming relevant baselines.

### 5.2. Limitations and Future Work

The current evaluation focused on low-noise settings with constant and limited rotational speeds. Future work should enhance domain adaptability and generalization so that high accuracy is maintained under varying speeds, loads, and temperatures. Approaches include transfer learning and domain adversarial networks (DANs). In addition, we used kurtosis—a linear statistic—for component selection; however, for nonlinear, non-stationary AE signals, critical information may reside in components with low kurtosis but high complexity. Future studies may combine nonlinear dynamical metrics such as sample entropy and permutation entropy with kurtosis in a multi-criterion selection scheme to better preserve essential fault characteristics, reduce dimensionality, and improve computational efficiency.

There is a trade-off between model complexity and real-time performance. While BiLSTM improves accuracy, its bidirectional processing introduces latency. To meet industrial real-time requirements, future work will explore lightweight architectures—such as 1D CNNs with attention—and model compression through quantization and pruning to enable efficient, low-latency diagnosis on edge devices.

## Figures and Tables

**Table 1:** The 6001 deep groove ball bearing geometric parameters.

Parameter	Value	Description
Inner diameter	12 mm	Bearing bore diameter
Outer diameter	28 mm	Bearing outer diameter
Width	8 mm	Bearing thickness
Pitch circle diameter	24 mm	Diameter of the circle passing through the centers of the rolling elements
Rolling element diameter	4.76 mm	Steel ball diameter
Number of rolling elements	8	Number of steel balls
Contact angle	0°	Deep groove ball bearing characteristics

**Table 2:** Network parameters of the proposed model.

Network Layer	Dimension	Stride	Number of Kernels	Output Dimension
Conv1	1 × 64	1 × 1	10	2048 × 16
MaxPool1	2 × 1	2 × 1	10	1024 × 16
Conv2	1 × 4	1 × 1	32	1024 × 32
MaxPool2	2 × 1	2 × 1	32	512 × 32
Conv3	1 × 4	1 × 1	64	512 × 64
MaxPool3	2 × 1	2 × 1	64	256 × 64
Conv4	1 × 4	1 × 1	128	256 × 128
MaxPool4	2 × 1	2 × 1	128	128 × 128
BiLSTM	-	-	35	512 × 1
Dropout	-	-	-	512 × 1
FullyConnected	-	-	5	5
Softmax	-	-	-	5

**Table 3:** Dataset division.

No.	Type	Groups	Sample Length	Training Samples	Test Samples	Tags
1	Inner ring fault	3	2048	90	30	1
2	Outer ring fault	3	2048	90	30	2
3	Ball fault	3	2048	90	30	3
4	Cage fault	3	2048	90	30	4
5	Combined fault	3	2048	90	30	5

**Table 4:** Model comparison.

Model	Accuracy (%)	Precision (%)	Recall (%)	F1-Score (%)	Standard Deviations
CNN–LSTM	78.67	77.50	79.83	78.63	±2.10
CNN–BiLSTM	85.33	84.20	86.45	85.03	±1.85
ICEEMDAN–CNN–LSTM	92.67	91.80	93.53	92.63	±1.50
ICEEMDAN–CNN–BiLSTM	98.00	97.53	98.62	98.00	±0.80

## Data Availability

Data are contained within the article.
